# Randomized Controlled Trial Comparing Autologous Protein Solution to Hyaluronic Acid Plus Triamcinolone for Treating Hip Osteoarthritis in Dogs

**DOI:** 10.3389/fvets.2021.713768

**Published:** 2021-07-28

**Authors:** Samuel P. Franklin, Ashley L. Franklin

**Affiliations:** ^1^Franklin Research and Innovation, Monument, CO, United States; ^2^Colorado Canine Orthopedics, Colorado Springs, CO, United States

**Keywords:** autologous protein solution, corticosteroid, hyaluronic acid, canine, hip dysplasia, osteoarthritis

## Abstract

Twenty-three dogs with bilateral hip osteoarthritis were used to compare the efficacy of intra-articular injections of autologous protein solution (APS) to hyaluronic acid plus triamcinolone (HAT). Prior to treatment, owner assessments of pain and mobility were obtained using the canine brief pain inventory (CBPI) and Liverpool Osteoarthritis for Dogs (LOAD) questionnaires. Owners were also asked to list all medications used to control signs of pain associated with hip osteoarthritis (OA). In addition, objective kinetic data using a pressure sensitive walkway was used to quantify the relative weight bearing of each of the limbs (total pressure index; TPI). One hip was then selected using a random number generator for injection with HAT and the contralateral hip was injected with APS under the same sedation event. At 1-, 3-, and 6 months following injection, medication usage was recorded and dogs were re-assessed using the CBPI and LOAD questionnaires and using objective gait analysis to determine the TPI. Twenty dogs completed all aspects of the study and statistically significant (*p* < 0.05) improvements were noted by dog owners at every post-treatment time point in every category of pain and mobility as assessed by the CBPI and LOAD questionnaires. Only 5 dogs, compared to 14 pre-treatment, received any oral NSAID or other analgesic for the duration of the 6-month study period. The TPI, and change in TPI from baseline, were not statistically significantly different between the two treatments at any time point. These data suggest clinical efficacy of both APS and HAT, but fail to show superiority of one treatment vs. the other. The inability to detect a statistically significant difference between the two treatments could be attributable to a true lack of a difference, or a type II statistical error.

## Introduction

Autologous protein solution (APS) is an autologous blood product involving preparation of leukocyte-rich platelet-rich plasma (L-PRP) from a single centrifugation cycle followed by desiccation with polyacrylamide beads. In horses and people, this preparation process results in a bioactive milieu with a favorable concentration of anti-inflammatory proteins, such as interleukin-1 receptor antagonist protein (1–3). No similar studies have been performed quantifying protein content of canine APS. Multiple prospective, randomized studies have shown superiority of APS over control when treating OA in horses, dogs, and people. Specifically, two placebo-controlled studies have shown significantly greater improvements in patient-reported outcomes in people with knee OA treated with APS in comparison to those patients treated with saline (4, 5). Likewise, a study in horses compared APS to saline and objective kinetic outcome data obtained with a force plate showed superiority of APS (1). Similarly, two studies using objective kinetic outcome data confirm superior results with intra-articular injection of APS when compared to saline in dogs with naturally-occurring OA (6, 7). In total, these data are consistent in showing that intra-articular injection with APS results in superior outcomes in multiple species when compared to saline controls.

Two of the unanswered questions with regard to APS use in dogs pertain to the duration of benefit, and whether APS provides a greater improvement than alternative injectable treatments such as hyaluronic acid and/or corticosteroid. This latter question is relevant because hyaluronic acid and corticosteroid are available “off-the-shelf” and are typically less expensive than APS. As for duration of benefit, the previous studies in dogs have followed patients just 1- and 3- months post injection, and so whether APS provides a longer benefit in dogs is unclear (6, 7). Two studies in people have shown superiority of platelet-rich plasma to steroid injection at mid or longer time frames (8, 9). In addition, one study in people showed improvements in self-reported outcomes up to 3 years post a single injection of APS (4). The most similar data in dogs was a study comparing platelet-rich plasma to hyaluronic acid plus corticosteroid for treating elbow OA (10). Dogs were followed for 6 months and benefits with both treatments were identified using validated owner questionnaires, but no objective kinetic outcome data were obtained. As a result, the questions as to superiority of APS to hyaluronic acid and steroid, as well as duration of APS benefit, remains unclear in dogs.

The purposes of the current study were to compare APS to hyaluronic acid combined with triamcinolone in dogs with naturally-occurring, bilateral hip osteoarthritis. Further, we sought to assess outcomes with a longer follow-up time than has been used previously when assessing dogs treated with APS. Based on aforementioned data in people, horses, and dogs, we hypothesized that there would not be a significant difference between the two treatments at first follow-up (1 month), but that the benefit of APS would be superior to that of HAT at subsequent follow-ups including up to 6 months post treatment.

## Methods and Materials

### Enrollment Criteria and Screening

With approval by the Animal Care and Use Committee of Colorado Canine Orthopedics and Rehab, and based upon a previous pilot study in which we compared APS to saline in dogs with hip OA using the same study methodology (6), we sought to enroll 20 dogs between 22 and 55 kg in body weight and between 2 and 10 years of age with bilateral hip osteoarthritis and without osteoarthritis of their shoulders, elbows, or stifles. All dogs were required to have no other medical conditions and to be off all medications and supplements for at least 7 days prior to enrollment and could not have received any intra-articular injections for 12 months prior to enrollment. Screening included consultation with the owner to discuss history, medical problems, and medication usage. In addition, a physical examination and subjective gait assessment were performed by the principal investigator (SPF), and radiographs were made of the hips, stifles, shoulders, and elbows. Specifically, medial-lateral radiographs were made of the shoulders, elbows, and stifles, and a lateral view of the pelvis was made. A cranial-caudal radiograph was made of both elbows simultaneously and a ventro-dorsal radiograph of the pelvis that included both stifles in the frontal plane were made. Any radiographic or physical exam evidence of pathology of any joints other than the coxofemoral joints resulted in exclusion of the patient from the study. Asymmetry in gait based upon the investigator's subjective gait assessment or the owner's history resulted in exclusion as the goal was to enroll symmetrically affected dogs.

### Baseline Data Collection

Following screening, dogs that met the inclusion criteria were enrolled and all owners provided written, informed consent for inclusion in the study. Prior to treatment owners then completed the Canine Brief Pain Inventory (CBPI) and the Liverpool Osteoarthritis in Dogs (LOAD) questionnaires. Dogs were also trotted across a pressure sensitive walkway (Gait4Dog^®^, CIR Systems, Franklin, NJ) to collect objective gait data. We sought to obtain a minimum of 5 valid trials. A trial was valid if the leash was slack and not influencing the dog's gait, the dog was trotting in a straight line while looking straight ahead without turning its head, and in which the dog maintained a relatively consistent pace. Dogs were typically trotted at least 20 times across the walkway in order to ensure obtaining five or more acceptable trials. All trials were video-recorded for subsequent review.

### APS Treatment

After collection of baseline data dogs were sedated with 5 μg/kg of Dexmedetomine (Zoetis, Kalamazoo, MI, USA) and 0.2 mg/kg of Butorphanol (Torbugesic, Zoetis, Kalamazoo, MI, USA) administered intravenously. An area over the jugular vein was then clipped and aseptically prepared and an 18-gauge 2″ IV catheter was then placed in the jugular vein. A 60 ml syringe that was pre-loaded with 5 ml of ACD-A (Citra Labs, Braintree, MA) was then attached to the catheter and filled with blood to its full 60 ml volume. The syringe was then gently inverted multiple times to mix the blood and anti-coagulant. A small volume (0.5–1 ml) of such blood was placed in a small purple top tube with EDTA for performing a whole blood complete blood count (CBC) on an in-house blood analyzer (Element HT5, Heska, Loveland, CO). It is worth noting that no validation of methods for counting cellular components, including platelets, in canine autologous protein solution specifically, has been performed.

The remaining blood was then used to prepare APS according to manufacturer instructions (Pro-Stride^®^ APS device, Owl Manor, Warsaw, IN). Following APS preparation 1 ml of APS was collected for administration to the patient and ~0.5–1 ml was taken for a CBC on the APS.

One hip was then selected for injection with HAT using a random number generator. The injection site was aseptically prepared, joint fluid was aspirated to confirm intra-articular needle placement and then the joint was injected with Triamcinolone (0.2 mg/kg; Kenalog-10, Bristol-Myers Squibb Company, Princeton, NJ, USA) combined with 1 ml of a medium molecular weight hyaluronic acid (Hyvisc^®^, Anika Therapeutics Inc., Bedford, MA). The patient was then rolled into the opposite recumbency, the contralateral hip was aseptically prepared, joint fluid aspirated, and 1 ml of APS was injected. Following treatment dogs were reversed from their sedation using Atipamezole (Revertidine, Modern Veterinary Therapeutics, Miami, FL, USA; equal volume as the dexmedetomidine; given intramuscularly) and discharged to the owners. Owners were provided 2 days of a non-steroidal anti-inflammatory drug (Carprofen; Vetoquinol, Ft. Worth, TX, USA) to use in the first 2 days following injection as experience has shown that many owners complain of patient discomfort in the first 2 days. Owners were also provided a daily log into which they recorded any adverse events or use of any medications or supplements. Owners were informed that they were allowed to use medications or supplements if necessary but were requested to contact the principal investigator prior to initiating using of any medications or supplements. All dogs were allowed to resume activity without limitation.

### Post Treatment Data Collection

At 1-, 3-, and 6 months following treatment dogs were re-evaluated. The principal investigator consulted with the owners, reviewed the owner logs, and recorded information regarding any adverse events and use of medications. Owners then repeated the CBPI and LOAD questionnaires without having access to prior CBPI and LOAD questionnaires. The same owner completed the CBPI and LOAD questionnaires at all time points. Dogs were then trotted across the pressure sensitive walkway as they had been prior to treatment so that TPI could be quantified.

## Data Evaluation and Statistical Analyses

### Radiography

Radiographs of the shoulders, elbows, hips, and stifles were evaluated by the principal investigator at the time of screening and enrollment to determine eligibility for enrollment. The hips were later re-evaluated by the principal investigator at least 9 months following treatment while blinded to treatment allocation. Hips were graded using the Orthopedic Foundation for Animals grading classifications for dogs with hip dysplasia as having either mild, moderate, or severe dysplasia.

### Objective kinetic Data

Videos of dogs trotting across the pressure sensitive walkway were reviewed. If any trial met the aforementioned criteria for a valid trial, the trial was quantitatively evaluated to obtain information on the relative weight bearing of each limb (total pressure index; TPI). The percentage of all 4 limbs adds up to 100% and a normal distribution is typically about 30% on each forelimb and 20% on each hindlimb. Only those trials in which there was a minimum of 2 full gait cycles and <10% variation in the dog's velocity were included.

Once the gait data were quantified the TPI of the two pelvic limbs were exported for assessment. The TPI and the change in TPI from baseline were selected as the outcome variable of interest before the study was conducted or the data analyzed. These data (TPI and change in TPI from baseline) were evaluated using a linear mixed model (LMM). The LMM included fixed factors for treatment and the time of the assessment (i.e., 0, 1, 3, 6 months) and a treatment by time interaction effect. The LMM also included random intercepts for each dog and each limb to account for within dog and within limb correlations. Satterthwaite degrees of freedom method was used. F-tests of simple treatment effects at each time point were performed. Model residuals were examined to evaluate the assumption of normality. These analyses were performed using SAS V 9.4 (Cary, NC).

### CBPI

The CBPI is a validated owner questionnaire for assessing canine pain and consists of 4 questions in which the owner assesses the dog's pain and six questions that evaluate the dog's function. For each of these questions, owners can provide an answer from 0 to 10 with 0 being consistent with a normal dog and 10 being consistent with either more pain or decreased function. The total scores for the first 4 questions (i.e., pain) were summed for each dog both prior to and at each time point (1, 3, and 6 months) following treatment as has been done in previous study (6). Similarly, the total scores for the six questions assessing function were summed for each dog both prior to and following treatment. Finally, there was one last question assessing “overall impression” that could be scored poor, fair, good, very good, or excellent by the owner. We changed these responses to this question to an ordinal scale (1–5) with 1 being poor and 5 being excellent, as was done in a previous study (6). For each of these three parts of the CBPI (pain, function, and overall impression) a LMM was used to assess change in scores over time. Model pairwise comparisons were adjusted for using a Dunnett's test.

### LOAD

The LOAD is another validated owner questionnaire for assessing canine mobility and has 5 questions that assess “mobility generally” and 8 questions that assess “mobility at exercise.” Each of these 5 questions allowed 5 discrete responses by the owner. For example, for the answer to the question regarding general mobility the owners could select very good, good, fair, poor, or very poor. We converted the responses for all questions into an ordinal scale (1–5) with higher scores consistent with decreased mobility, as done in previous study (6). As for the CBPI, the total score for the 5 questions assessing “general mobility” were summed for each dog both prior to and following treatment. The 8 questions assessing “mobility at exercise” were similarly summed for each dog both prior to and following treatment. For each of these two parts of the LOAD (“general mobility” and “mobility at exercise”) a LMM was used to assess change in scores over time. Model pairwise comparisons were adjusted for using a Dunnett's test.

### *Post-hoc* Assessments

Correlation was assessed *post-hoc* between the change in TPI (from baseline to 3 months) in the APS-treated limb and both the leukocyte concentration and the platelet concentration in the APS using a Pearson product moment correlation.

The mean TPI in the APS-treated limb at 3 months post treatment was compared between dogs that did or did not receive any carprofen following treatment (excluding the first 48 h following injection), using a *T*-test (unpaired, 2-tailed, significance set at *p* < 0.05).

The CBPI and LOAD scores at 3 months for pain, function, overall function, general mobility, and mobility at exercise were all compared between dogs that did or did not receive any carprofen following treatment using *T*-tests (unpaired, 2-tailed, significance set at *p* < 0.05).

The 3-month time frame was chosen for these *post-hoc* assessments because as shown below (see the results and **Figure 3**), month 3 was when the APS treated limbs had shown their greatest improvement (based on change in TPI) and were at their best (greatest TPI).

## Results

### Patient Demographics

Twenty-three dogs met the inclusion criteria and were enrolled. Twenty dogs completed the study and 3 were removed from the study. The 20 dogs that completed the study included 4 German shepherd dogs, 3 each of border collies, Labrador, and Golden retrievers, and one Australian shepherd, Doberman, Irish setter, Poodle mix, Rottweiler, Siberian husky, and St. Bernard mix. Ten dogs were spayed females, nine were castrated males, and there was one intact female. The mean weight was 33.62 kg (stdev 6.8 kg). The mean age was 4.8 years (2.2).

Both hips were classified as having mild hip dysplasia in 1 dog, moderate hip dysplasia in 2 dogs, and severe hip dysplasia in 15 dogs. In two dogs one hip was graded as severe and the contralateral was graded as moderate hip dysplasia. In one of these two dogs the randomization resulted in the hip with moderate dysplasia being treated with APS and in the other dog the hip with moderate dysplasia was treated with HAT based on the random number generator.

The 3 dogs that were removed from the study included a 2-year-old Husky that was attacked by another dog <4 weeks after treatment and had wounds to the left forelimb and associated left forelimb lameness at the 4-week recheck, thus precluding accurate objective assessment of the relative weight bearing on each of the hindlimbs. A 2-year-old German Shepherd dog developed myasthenia gravis 2 months following treatment, hampering his ability to walk and thus precluding assessment of gait. An 8-year-old castrated male German Shepherd dog developed a septic joint and polymyositis within 48 h following treatment. All data from these three dogs were omitted from all analyses.

### Medication Usage

Prior to study enrollment 5 dogs were not receiving any oral medications or supplements to help control pain or lameness attributable to hip osteoarthritis. One dog was treated with cannabidiol oil only. Fourteen of 20 dogs were receiving carprofen prior to study enrollment. Of these 14 patients receiving carprofen, eight were also receiving a glucosamine chondroitin supplement, four were being treated with Gabapentin, three were receiving fish oil supplementation, one was receiving Tramadol, and one was receiving Grapiprant.

All these medications and supplements were discontinued a minimum of 1 week prior to treatment and all owners were provided with 2 days of carprofen to use in the 2 days immediately following treatment. After this 2-day period of carprofen use, five dogs received carprofen for 23 cumulative days (mean 4.6 days/dog) over the subsequent 6 months. One of those dogs accounted for more than half (12/23 days) of the carprofen usage with the remaining 4 dogs averaging fewer than 3 days of carprofen use over the 6 months. One of these dogs also received 3 days of Gabapentin concurrent with the carprofen. One additional dog didn't receive any caprofen but the owner re-started glucosamine and chondroitin 3 months following treatment.

### Adverse Events

Six owners reported perceived discomfort following injections that resolved by 48 h post injection in all cases.

One aforementioned patient developed a septic arthritis and septic polymyositis following APS injection. Bacterial culture of fluid obtained from the affected joint and the vastus lateralis both confirmed infection with *Escherichia coli*. The patient was treated with two rounds of hypodermic needle lavage of the affected coxofemoral joint, ultrasound-guided drainage of intramuscular pockets of fluid in the vastus lateralis, and oral and intravenous antibiotics and the infection resolved.

### Complete Blood Count Data

The results of the CBCs showed a mean increase of 11.1 times (stdev 2.1) the leukocytes in the APS in comparison to the baseline whole blood. There was a mean increase of 1.4 times (1.1) the platelets in the APS when compared to the whole blood.

### Owner Assessment Data

#### CBPI

**Figure 1:**
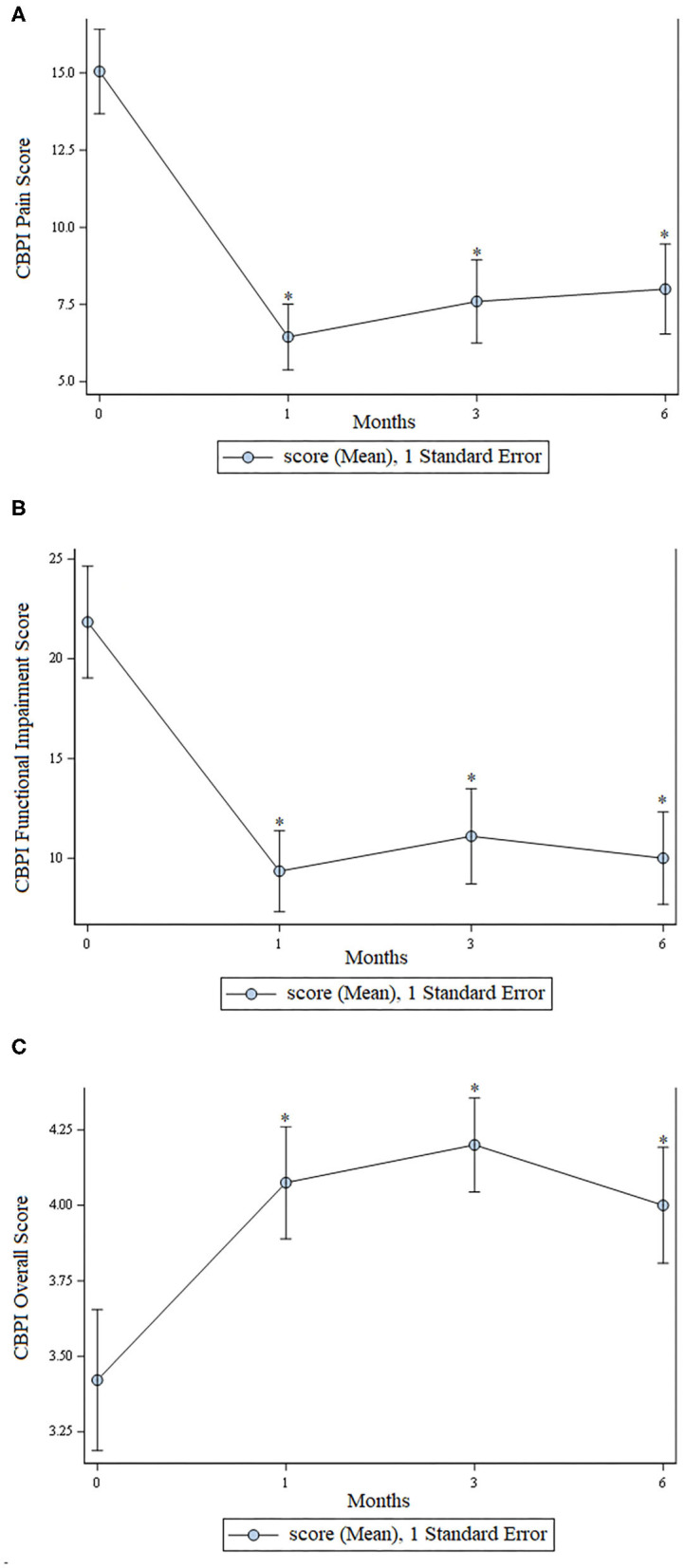
Scores from the Canine Brief Pain Inventory. **(A)** Pain scores; lower scores indicate less pain. **(B)** Functional impairment; lower scores indicate less functional impairment. **(C)** Overall owner assessment; higher numbers are better. In all graphs an * indicates a significant (*p* < 0.05) change from baseline.

There was a statistically significant improvement (*p* < 0.01) at all time points for assessment of pain, function, and overall score as assessed using the CBPI. These data are shown in Figure 1.

#### LOAD

**Figure 2:**
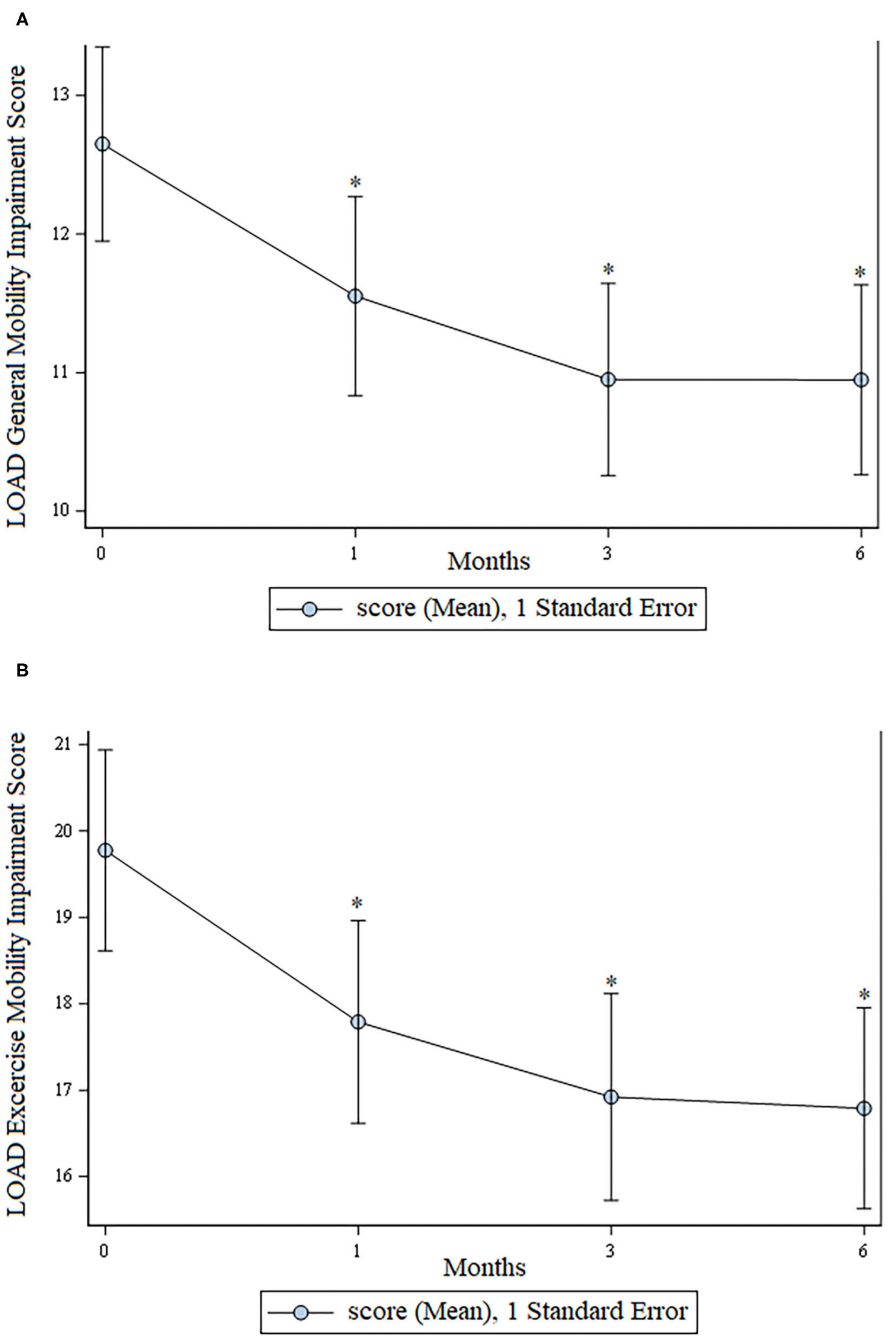
LOAD scores. **(A)** Mobility in general; **(B)** Mobility at exercise. For both graphs, lower scores are consistent with greater mobility. In both graphs an * indicates a significant (*p* < 0.05) change from baseline.

There was a statistically significant improvement (*p* < 0.05) at all time points for assessment of general mobility and mobility at exercise as assessed using the LOAD questionnaire. These data are shown in Figure 2.

### Total Pressure Index

**Figure 3:**
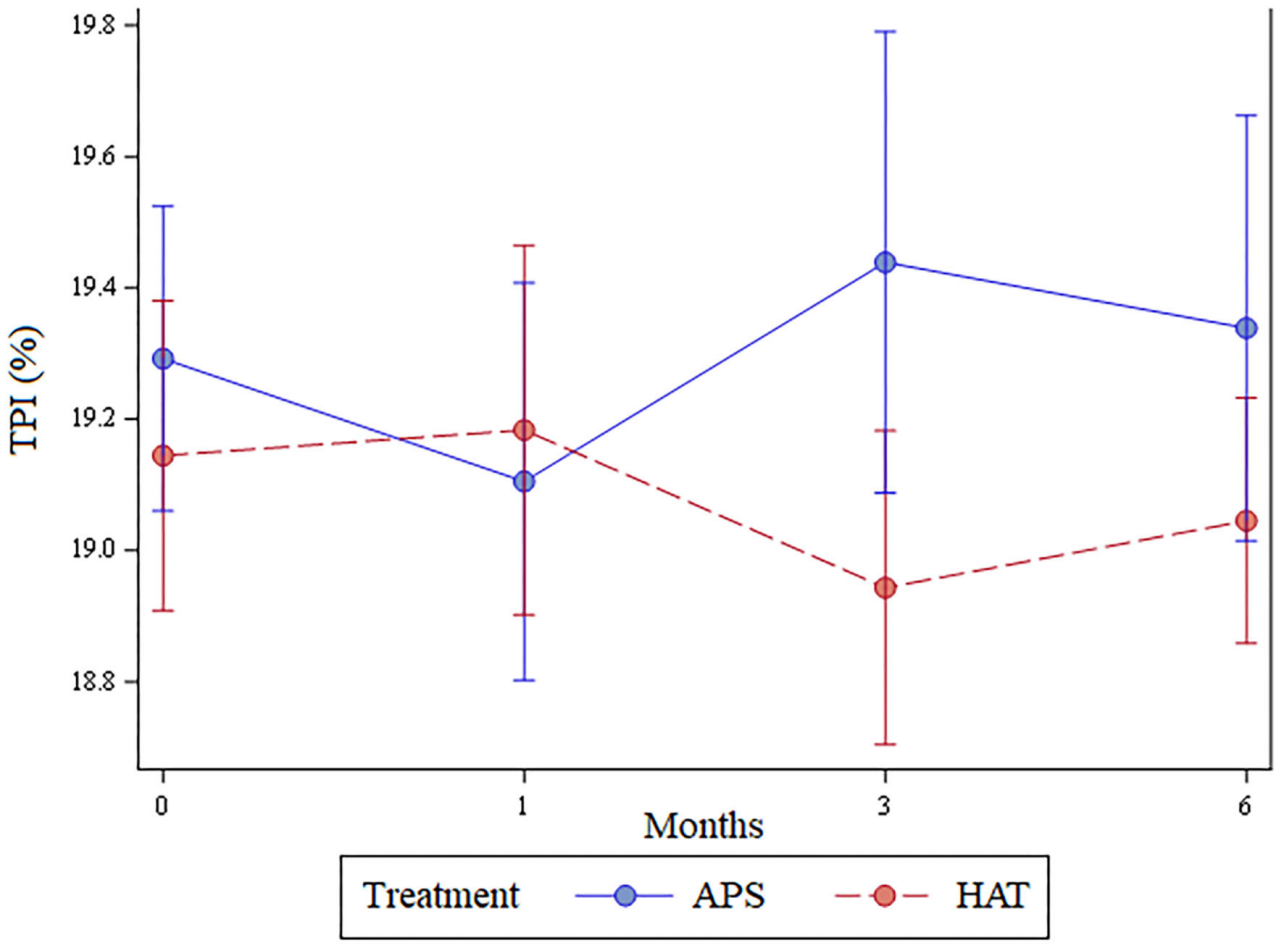
Total pressure index (mean ± SE) over the 6-month study for APS and HAT treated limbs.

**Figure 4:**
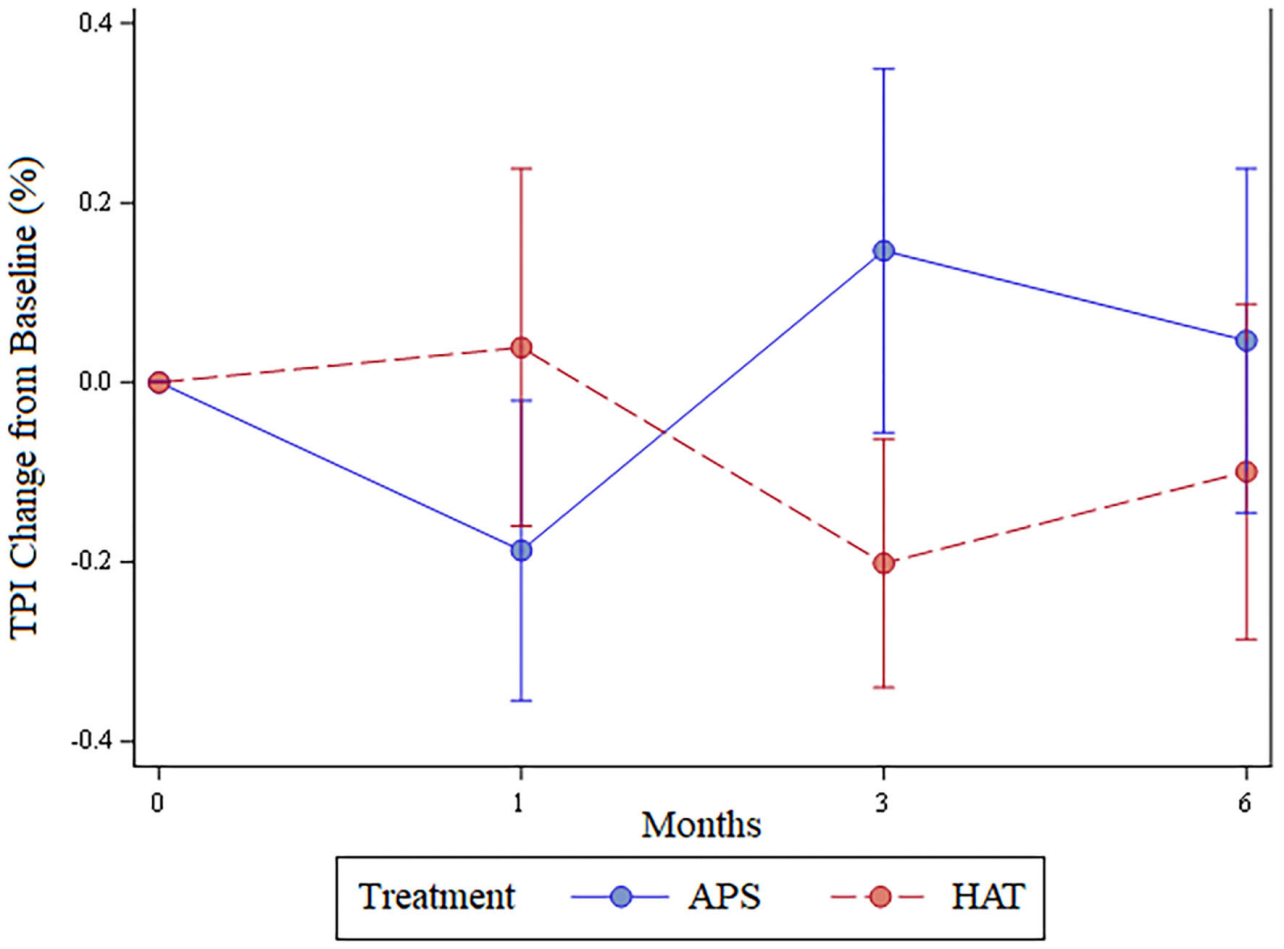
Change in total pressure index (mean ± SE) from baseline over the 6-month study for APS and HAT treated limbs.

The least number of valid trials obtained and used in statistical analyses for any dog at any time point was 6. The TPI was not significantly different between limbs pre-treatment (*p* = 0.62), at 1 month (*p* = 0.7), 3 months (*p* = 0.22), or 6 months (*p* = 0.44); Figure 3. The change in TPI from baseline was not statistically significantly different between treatment limbs at 1 month (*p* = 0.36), 3 months (*p* = 0.16), or at 6 months (*p* = 0.44; Figure 4). Change in TPI for each limb was related in that for every time point that TPI increased for the APS-treated limbs, the TPI decreased for the HAT-treated limbs, and vice versa (see Figures 3, 4).

### *Post-hoc* Comparisons

There was no statistically significant correlation (*p* > 0.05) between the TPI at 3 months in APS treated limbs, or the change in TPI between 0 and 3 months in APS-treated limbs, and either the leukocyte concentration or the platelet concentration in the APS.

There was no statistically significant difference (*p* = 0.59) in the TPI at 3 months in APS treated limbs for dogs that did or did not receive any carprofen. Similarly, there were no statistically significant differences (*p* > 0.05) in CBPI or LOAD scores at 3 months post injection between dogs that did or did not receive carprofen in the 6 months following treatment.

## Discussion

The owners consistently perceived their dogs as being substantially improved for the entire 6- month duration of the study. This is supported by the fact that scores for all 5 categories of pain, function, and mobility using the CBPI and LOAD questionnaires were statistically significantly improved at all post-treatment time points when compared to baseline. In addition, this owner-perceived improvement was concurrent with a substantial reduction in use of oral medications including non-steroidal anti-inflammatory drugs. Fourteen dogs were receiving an NSAID prior to study enrollment and only 5 received an NSAID during the 6 months following, with all but one doge receiving <3 days of NSAID therapy during the 6 months following treatment.

Since a previous study has shown an owner caregiver placebo effect of 40% (11), we believe that caregiver placebo effect influenced these aforementioned results, including the subjective questionnaire data and also possibly the NSAID administration data. However, we doubt that the improvements noted are only attributable to caregiver placebo and suspect that there was likely veritable improvement for two reasons. First, the improvements in the CBPI and LOAD were consistent and substantial. More importantly, as detailed in the introduction, numerous previous studies in different species, including two previous studies in dogs with objective, kinetic outcome data have proved that APS provides veritable improvements in comparison to saline control (6, 7). Likewise, at least one, well-controlled study in research dogs showed statistically superior TPI in dogs treated with hyaluronic acid in comparison to saline controls (12). Therefore, we suspect that patients were improved because each treatment had some beneficial effect for each hip. However, it is important to note that the data from this study alone do not enable us to definitively draw the conclusion that the dogs improved over time.

As for the difference in treatment efficacy between APS and HAT, the study was designed specifically to assess this question with the total pressure index being the sole outcome measure for such comparison. The data failed to show a statistically significant difference between the two treatments, either because no difference exists, or because there was a type II statistical error. We cannot differentiate between these two potential explanations and the first potential explanation is possible for numerous reasons. The study was designed to optimize the likelihood of detecting a statistically significant difference between these two treatments. We treated the same dogs with two different treatments simultaneously and then compared the relative weight bearing of the two limbs objectively. With this methodology there is effectively no inter-dog treatment difference that need to be accounted for because all dogs receive both treatments. Similarly, there is not inter-trial variation associated with different trials across the pressure sensitive walkway that needs to be accounted for because both treatments are being assessed on each trial across the walkway. In turn, these study characteristics minimize the number of dogs needed to detect a statistically significant difference between two treatments. Furthermore, we met our pre-determined study numbers in terms of enrolling and obtaining complete data from 20 dogs, which we suspected a priori would be enough to detect a statistical difference. This presumption had been based in part on estimates of variability in a previous and similarly conducted study in which APS was found to provide superior results to saline using just 5 dogs with bilateral hip OA (6). As a result, it is possible that no clinically relevant difference exists between the two treatments tested in this study over a 6-month period in regards to improved lameness.

While the aforementioned conclusion that there is no significant difference might be true, it is also possible that we had a type II statistical error. Numerically, but not statistically significantly, the hips treated with HAT had a greater TPI at 1 month post treatment while the APS-treated limbs had a greater TPI at 3- and 6 months post treatment (Figure 3). At least two previous studies in people have shown a greater benefit with PRP compared to steroid at 12 months following treatment and so we had hypothesized that the APS would be of greater benefit than HAT later in the study (8, 9). The data fit this pattern numerically, but not significantly. As a result, it is possible that a difference does exist that we might have detected had we included more dogs. It should also be noted that the results could be dependent upon or influenced by the breed, joints, severity of the OA, relative exercise levels or purpose of the dog, and age, weight, or gender. Future studies could potentially be designed to assess the impact of these characteristics on the results.

There were some associated limitations of the study design, most notably that the pressure sensitive walkway used does not provide an absolute value for weight-bearing in the form of peak vertical force or vertical impulse. Rather, the relative weight distribution of each limb is provided (the TPI) and the TPI of one limb is affected by the weight bearing of the contralateral limb. This phenomenon is easily visualized in Figure 4. The change in TPI of one hindlimb was always the opposite of the contralateral; as the TPI increased for APS-treated limbs the TPI decreased for HAT-treated limbs. Coupled with owner assessments of the dog, rather than owner assessment of individual limbs, we cannot state whether absolute weight bearing of either of the two limbs increased post treatment or not. Rather, we have to return to our previously stated conclusions that owner-based assessments, which are subject to caregiver placebo effect, showed that the dogs improved over time and that we hypothesize the dogs likely improved in weight bearing over time based in part on previous studies showing efficacy of these treatments in comparison to saline. However, the data from this study cannot definitely prove that the dogs improved with treatment. Further, there was no statistically significant differences between the two treatments assessed in this study and we cannot determine if that is the result of a type II statistical error or lack of a true difference between the two different treatments assessed.

## Data Availability Statement

The raw data supporting the conclusions of this article will be made available by the authors, without undue reservation.

## Ethics Statement

The animal study was reviewed and approved by Colorado Canine Orthopedics and Rehab Animal Care and Use Committee. Written informed consent was obtained from the owners for the participation of their animals in this study.

## Author Contributions

SF designed the study, executed data collection, and wrote the manuscript. AF assisted with study coordination, data collection, patient treatment, and manuscript revision. Both authors contributed to the article and approved the submitted version.

## Conflict of Interest

SF and AF are owner/operators/employees of Franklin Research and Innovation.

## Publisher's Note

All claims expressed in this article are solely those of the authors and do not necessarily represent those of their affiliated organizations, or those of the publisher, the editors and the reviewers. Any product that may be evaluated in this article, or claim that may be made by its manufacturer, is not guaranteed or endorsed by the publisher.
